# Association between Coffee Consumption and Its Polyphenols with Cardiovascular Risk Factors: A Population-Based Study

**DOI:** 10.3390/nu9030276

**Published:** 2017-03-14

**Authors:** Andreia Machado Miranda, Josiane Steluti, Regina Mara Fisberg, Dirce Maria Marchioni

**Affiliations:** Department of Nutrition, School of Public Health, University of São Paulo, São Paulo 01246-904, Brazil; jsteluti@gmail.com (J.S.); rfisberg@usp.br (R.M.F.); marchioni@usp.br (D.M.M.)

**Keywords:** coffee consumption, coffee polyphenol intake, cardiovascular risk factors, representative sample

## Abstract

Epidemiological studies have examined the effect of coffee intake on cardiovascular disease, but the benefits and risks for the cardiovascular system remain controversial. Our objective was to evaluate the association between coffee consumption and its polyphenols on cardiovascular risk factors. Data came from the “Health Survey of São Paulo (ISA-Capital)” among 557 individuals, in São Paulo, Brazil. Diet was assessed by two 24-h dietary recalls. Coffee consumption was categorized into <1, 1–3, and ≥3 cups/day. Polyphenol intake was calculated by matching food consumption data with the Phenol-Explorer database. Multiple logistic regression models were used to assess the associations between cardiovascular risk factors (blood pressure, total cholesterol, low-density lipoprotein cholesterol (LDL-c), high-density lipoprotein cholesterol (HDL-c), triglycerides, fasting glucose, and homocysteine) and usual coffee intake. The odds were lower among individuals who drank 1–3 cups of coffee/day to elevated systolic blood pressure (SBP) (Odds Ratio (OR) = 0.45; 95% Confidence Interval (95% CI): 0.26, 0.78), elevated diastolic blood pressure (DBP) (OR = 0.44; 95% CI: 0.20, 0.98), and hyperhomocysteinemia (OR = 0.32; 95% CI: 0.11, 0.93). Furthermore, significant inverse associations were also observed between moderate intake of coffee polyphenols and elevated SBP (OR = 0.46; 95% CI: 0.24, 0.87), elevated DBP (OR = 0.51; 95% CI: 0.26, 0.98), and hyperhomocysteinemia (OR = 0.29; 95% CI: 0.11, 0.78). In conclusion, coffee intake of 1–3 cups/day and its polyphenols were associated with lower odds of elevated SBP, DBP, and hyperhomocysteinemia. Thus, the moderate consumption of coffee, a polyphenol-rich beverage, could exert a protective effect against some cardiovascular risk factors.

## 1. Introduction

Cardiovascular diseases (CVD) are considered to be the leading global cause of death, accounting for 17.3 million deaths per year, which is predicted to rise to more than 23.6 million by 2030 [1]. The main causes of CVD involve non-modifiable risk factors, in addition to the metabolic risk factors, that are targeted together with the behavioral risk factors, such as unhealthy diets (rich in salt, saturated fat, and calories) [2]. However, there are still food items whose role is controversial, such as coffee.

Coffee has been considered an important dietary factor, because it is one of the most popular and widely consumed nonalcoholic beverages in the world. Finland is the largest coffee consumer market, followed by Brazil. In Brazil, the average coffee consumption is 5.9 kg per capita [3], with an estimated prevalence of intake of 79%, i.e., the second-most consumed food in the country [4].

Coffee beverage, a mixture of several pharmacologically-bioactive compounds, including caffeine, phenolic acids, and the diterpene alcohols, cafestol and kahweol, can also have long term effects on risk factors for CVD, such as blood pressure, plasma concentrations of cholesterol and homocysteine, and the incidence of type 2 diabetes mellitus [5,6,7].

Caffeine, a central nervous system stimulant and psychoactive substance, has been positively associated with blood pressure [8,9], systemic vascular resistance and unfavorable effects on endothelial function [9], serum lipids concentration [10], and insulin resistance [11]. Other prospective studies, however, have generally not supported adverse risk effects on CVD associated with coffee consumption [12,13,14]. The major beneficial properties of coffee seem to depend on its content of phenolic acids, which demonstrates protective roles in the cardiovascular system [15]. This cardiovascular protection has been demonstrated in vivo, and can be explained by various mechanisms, including their anti-inflammatory properties [16], the strong antioxidant capacity [17] related to nitric oxide (NO) bioavailability, as well as low-density lipoprotein (LDL) oxidation, and antithrombotic properties through endothelial protection [18]. 

Contrary to earlier studies focused on caffeine, existing evidence is suggesting that coffee may exert a beneficial effect toward cardiovascular-related outcomes, together with all-cause and cancer mortality [19]. However, the public debate about reducing or increasing the risk of CVD by drinking coffee is still relevant due to the previous contrasting findings on cardiovascular effects. Additionally, its effects on CVD might have considerable public health and clinical implications [12].

Therefore, the current study aimed to assess the association between usual coffee consumption and coffee polyphenol intake on cardiovascular risk factors, e.g., systolic and diastolic blood pressure, total cholesterol, low-density lipoprotein cholesterol (LDL-c), high-density lipoprotein cholesterol (HDL-c), triglyceride, fasting plasma glucose, and homocysteine, in a representative sample of individuals aged 20 years or older in São Paulo City. 

## 2. Materials and Methods

### 2.1. Study Population

Data were retrieved from the “Health Survey of São Paulo (ISA-Capital)”, a cross-sectional population-based study designed to assess the health and nutritional status of non-institutionalized individuals residing in São Paulo City in Southeastern Brazil, between 2008 and 2009. 

A complex probabilistic sampling, by conglomerates, based on census tracts and households that had already been drawn in the National Household Sample Survey 2005 (PNAD 2005) was used. The drawing was systematic, and eight study domains were defined: less than one year old; one to 11 years old, and three more age groups by sex: 12 to 19 years (adolescents), 20 to 59 years (adults), and 60 years or over (older adults). A minimum sample size of 300 in each of the six domains was estimated to be needed based on a prevalence of 0.5 with a standard error of 0.07 at a 5% significance level and a design effect of 1.5. 

A total of 2691 individuals, aged 12 years or over, were selected to answer questions about diet, life conditions, and sociodemographic information. Thereby, only 1662 individuals of the initial sample agreed to participate. Of those, 750 subjects provided a blood sample for biochemical analysis, completed two 24-h dietary recalls (24 HR), anthropometric data, as well as arterial blood pressure measurements. For the present study, only adults and older adults were included, totaling a final sample of 557 individuals.

The study protocol was reviewed and approved by the Ethics Committee at the School of Public Health, University of São Paulo (Approval Number: 003.0.162.000-08). A written informed consent form was obtained from all participants.

### 2.2. Dietary Assessment

The dietary intake was measured by two 24 HR. The first 24 HR was administered at households by trained interviewers using the multiple pass method [20]; the second 24 HR was performed by telephone using the automated multiple pass method [21]. The multiple pass method, automated or not, is structured in five steps: (1) a quick list, where participants list all of the foods and beverages consumed uninterruptedly; (2) a forgotten list, where participants are asked about commonly consumed forgotten foods, such as candies, coffees, and sodas; (3) time and location of food and beverage intake; (4) detailing cycle, that is, a description of the way of preparation and amounts consumed; and (5) a final review, which verifies whether a certain food consumed during the day was not previously recorded. The sampling days covered all the days of the week and seasons. Dietary data were entered into the Nutrition Data System for Research software (version 2007, University of Minnesota, Minneapolis, MN, USA), which is mainly based on data from the food composition table published by the United States Department for Agriculture (USDA).

The multiple source method (MSM), a statistical modeling technique, was used to estimate the usual dietary intake of polyphenols and nutrients and to remove within-person variation. In the first step, the probability of eating the food on a random day for each individual was estimated by a logistic regression model. Secondly, the usual amount of food or nutrient intake is estimated by a linear regression model. Finally, the resulting numbers from step one and two are multiplied by each other to estimate the usual daily intake for each individual [22].

#### 2.2.1. Assessment of Coffee Consumption

In the 24 HR, the participants reported if they consumed coffee on the day before the interview and, thereafter, a question about the method of coffee preparation (filtered, instant, espresso, or other), and whether additional items were typically added to the coffee (none, milk, sugar, artificial sweetener, etc.) were probed. Daily coffee intake (in mL) was categorized, according to the standard cup size used in the study (50 mL), into three categories: <1 cup/day, 1–3 cups/day, and ≥3 cups/day. The category of <1 cup/day of coffee was used as the reference group.

#### 2.2.2. Estimation of Polyphenol Intake from Coffee

Data on the polyphenol content in foods were obtained from the Phenol-Explorer database [23] that presents data on the content of 502 polyphenols in 452 foods [24].

The polyphenol intake was calculated by matching usual food intake data from the 24HR with the polyphenol content in foods from the Phenol-Explorer database. The individual polyphenol intake from each food was calculated by multiplying the content of each polyphenol by the daily consumption of each food, including coffee. The total polyphenol intake was the sum of all individual polyphenol intakes from all food sources reported in the 24 HR. Other details on the estimation of polyphenol intakes are available elsewhere in previous publications [25].

For the present study, the classes of polyphenols ingested from coffee included the phenolic acids, especially hydroxycinnamic acids (4-caffeoylquinic acid, 5-caffeoylquinic acid, 3-caffeoylquinic acid, 5-feruloylquinic acid, caffeic acid), alkylmethoxyphenols (4-ethylguaiacol, 4-vinylguaiacol), and others (catechol, pyrogallol, phenol). The coffee polyphenols were categorized into three categories: <101 mg/day (corresponding to <1 cup coffee/day), 101–337 mg/day (corresponding to 1–3 cups/day), and ≥337 mg/day (corresponding to ≥3 cups/day).

### 2.3. Demographic and Lifestyle Characteristics

Sociodemographic and lifestyle characteristics included age, sex, household per capita income, smoking status, alcohol drinking, and the use of medicines.

The physical activity included energy expenditure in leisure time by reporting type and duration of activity according to the predetermined questionnaire items of the long version of the International Physical Activity Questionnaire (IPAQ) [26], validated in Brazil. The physical activity level was categorized as daily low, moderate and high. In the current study, physical activity was grouped in two categories: low and moderate/high. More details for levels of physical activity proposed by IPAQ are available online [27].

The smoking status was categorized as nonsmoker and current smoker or former smoker.

### 2.4. Anthropometric Measurements

Anthropometric measurements were obtained in participant homes by a trained nursing assistant following the procedures recommended by the WHO [28]. Body weight (kg) was measured using a calibrated digital scale (Tanita^®^, model HD-313, Tanita Corporation of America, Inc., Arlington Heights, IL, USA). Height (cm) was measured with a portable wall-mounted stadiometer (Seca^®^, model 208, Seca Brazil, São Paulo, Brazil). Body mass index (BMI) was calculated by dividing weight (kilograms) by the square of the height (meters).

### 2.5. Outcome Measurements

#### 2.5.1. Blood Pressure (BP)

During the home visit, BP was measured using an automatic blood pressure monitor (Omron HEM-712C, Omron Health Care, Inc., Vernon Hills, IL, USA) handled by a nursing technician, according to the recommendations of the V Brazilian Guidelines on Hypertension [29]. Participants were considered to have high BP if they had systolic (SBP) and/or diastolic (DBP) higher or equal to 140 mmHg and 90 mmHg, respectively, according to the national and international recommendations [29,30].

#### 2.5.2. Blood Samples

The blood samples were collected by venipuncture after 12-h of overnight fasting by a nursing assistant, using a standardized protocol.

Serum total cholesterol (TC) and fractions, LDL-c and HDL-c, as well as triglyceride (TG), were determined by enzymatic-colorimetric method (Roche Diagnostics GmbH, Mannheim, Germany). We considered the cut-off point value for elevated TC level ≥200 mg/dL, for elevated LDL-c >100 mg/dL, reduced HDL-c <40 mg/dL in males or <50 mg/dL in females, and elevated TG ≥150 mg/dL [31].

Fasting plasma glucose (FPG) was measured by enzymatic-colorimetric glucose oxidase procedure using the kit gluco-quant Glucose/HK (GLU Roche/Hitachi 1,447,513; Roche Diagnostics GmbH, Mannheim, Germany). The cut-off value for elevated fasting glucose level was ≥100 mg/dL [31].

The immunoassay method of chemiluminescence microparticles using the ARCHITECT Homocysteine Reagent Kit (Abbott Diagnostics Division, Abbott Park, Lake Forest, IL, USA) was used to analyze the plasma concentrations of homocysteine. We selected the value of plasma homocysteine >16 μmol/L for older adults and a value >12 μmol/L for adults, as cut-off point for categorization in hyperhomocysteinemia [32].

### 2.6. Statistical Analysis

The characteristics of the study participants were described by coffee consumption categories, and presented as medians and interquartile range (IQR) for continuous variables, and frequencies and percentages for categorical variables. The variables were examined for normality (Kolmogorov-Smirnov test). Differences between coffee consumption categories were tested by Kruskall-Wallis test for continuous variables, and by the Chi-square test for categorical variables. 

The associations between the independent variables (categories of usual coffee consumption and coffee polyphenol intake) and the following dependent variables (i.e., SBP, DBP, FPG, serum TC, LDL-c, HDL-c, TG, and homocysteine) were tested by multiple logistic regression analysis.

Two models were fitted for each independent variable. The first model was the crude model (unadjusted). The second model (adjusted), corresponding to models 1, 2, 3, and 4, was adjusted for classic potential confounders (i.e., age, sex, race, BMI, smoking status, alcohol consumption, physical activity level, household per capita income, intake of caffeine, added sugars, total energy intake, and saturated fat), and additionally adjusted for particular variables hypothesized to be associated with each cardiovascular risk factor, according to the literature. Thus, model 1 corresponded to both SBP and DBP was additionally adjusted for sodium intake, and antihypertensive drugs; model 2 corresponded to fasting plasma glucose was additionally adjusted for hypoglycemic drugs; model 3 corresponded to TC, LDL-c, HDL-c, and TG was additionally adjusted for monounsaturated fat, polyunsaturated fat, and hypolipidemic medicines; and lastly model 4 corresponded to homocysteine was additionally adjusted for folate, B6, and B12 vitamins. 

All analyses were conducted using the appropriate sample weights to account for the complex survey design. For all analyses, Stata^®^ statistical software package version 12 (StataCorp LLC, College Station, TX, USA) was used and a *p*-value < 0.05 was considered statistically significant.

## 3. Results

The final study population had a mean age of 45.1 years, mostly women (54.2%), self-declared white (61.1%), non-smokers (58.4%), insufficiently active (77.7%), and non-obese (74.5%).

The mean of coffee consumption for the whole population was 143.4 mL/day. A minority of participants consumed espresso coffee (*n* = 7), and there were no decaffeinated coffee consumers in the current study population. Furthermore, the median total intake of polyphenols was 363.9 mg/day. Coffee provided 247.0 mg/day of polyphenols, which represented approximately 68% of the total intake.

The sociodemographic, biochemical, and dietary characteristics of the studied population according to coffee consumption categories are shown in Table 1. Coffee drinking was higher in older adults than in adults, and among current smokers than in non-smokers. Differences were also observed between the usual coffee consumption and polyphenols intake from coffee, and caffeine intake. A further significant difference by coffee consumption was found with prevalence of DBP.

The associations between coffee consumption categories and cardiovascular risk factors are presented in Table 2. The adjusted models demonstrated lower odds for SBP, DBP, and homocysteine in individuals that were consuming 1–3 cups of coffee per day, than in individuals who drank less than 1 cup of coffee per day to elevated SBP (Odds Ratio (OR) 0.45, 95% Confidence Interval (95% CI): 0.26, 0.78); elevated DBP (OR 0.44, 95% CI: 0.20, 0.98), and hyperhomocysteinemia (OR 0.32, 95% CI: 0.11, 0.93). For those subjects with higher consumption (≥3 cups/day), the association was not significant for any cardiovascular risk factors. 

Table 3 shows the association between the same cardiovascular risk factors and categories of coffee polyphenols in this population. After adjustment for potential confounding factors, OR showed that a moderate intake of coffee polyphenols (101 to 337 mg/day) was inversely associated with elevated SBP (OR 0.46, 95% CI: 0.24, 0.87), elevated DBP (OR 0.51, 95% CI: 0.26, 0.98), and hyperhomocysteinemia (OR 0.29, 95% CI: 0.11, 0.78).

## 4. Discussion

The current study found that a moderate coffee consumption (1 to 3 cups per day), which corresponds to a coffee polyphenol intake of 101–337 mg/day, had a beneficial effect on cardiovascular risk factors, such as, elevated SBP, elevated DBP, and hyperhomocysteinemia.

Previous epidemiological studies on the benefits of coffee consumption on the cardiovascular system have provided inconsistent and conflicting results [33]. In this way, some studies suggested a positive association between coffee consumption and risk of CVD [34,35], whereas others reported no association [36,37], or even an inverse association [12,13,14]. Therefore, these controversial findings may be due to the different types of studies, with different designs performed in distinct populations. Additionally, inconsistent and conflicting results may be related to the various confounding dietary factors, different forms of brewing coffee and the daily amount consumed.

Numerous risk factors for CVD have been described in the literature, and elevated BP is a recognized and well-established risk factor for coronary heart disease and stroke. A large number of epidemiological studies on the effect of coffee or caffeine on BP has been published, but they have provided conflicting results, and the effect of chronic coffee consumption on BP is still unclear [9,38]. A meta-analysis of randomized controlled clinical trials has concluded that regular coffee consumption slightly increases systolic and diastolic BP [8]. Interestingly, Noordzij et al. [8] showed that the BP elevations were larger in the studies using caffeine than in the studies on coffee consumption. In agreement with these findings, another recent systematic review and meta-analysis reports that BP elevations appeared to be significant only for caffeine but not for coffee consumption [9]. The prevailing explanation for such effect is that caffeine antagonizes endogenous adenosine, resulting in vasoconstriction and elevated total peripheral vascular resistance [39]. Moreover, although these results suggesting that caffeine acutely increasing BP, its effects may be somehow attenuated if ingested as coffee, so, it seems that other compounds in the coffee could potentially counterbalance the pressor effect of caffeine [8]. Coffee is a drink with a very complex chemical composition, rich in BP-lowering minerals (that is, potassium and magnesium) and antioxidant compounds (phenolic acids) that may outweigh the hypertensive effects of caffeine [40]. The beneficial effects of these other components in the cardiovascular system, may help explain the lack of association between long-term coffee consumption and increase BP or CVD risk in large cohort studies [9].

It was observed in our study that moderate coffee drinking was associated with lower odds of elevated SBP and DBP, especially due to the presence of polyphenols. The high content of phenolic compounds in coffee, especially due to the group of hydroxycinnamic acids (caffeic, chlorogenic, feluric, and p-coumaric acids), exhibits a strong antioxidant activity, and protects against atherothrombosis or atherosclerotic lesion development through endothelial protection (attenuates oxidative stress, improved NO bioavailability, and decreased E-selectin, intercellular adhesion molecule-1 (ICAM-1), and vascular cell adhesion protein-1 (VCAM-1) expression, among others) [18,41]. In this context, investigations during the last decade have implied that chlorogenic acid consumption can have a significant lowering effect on BP [42,43]. This hypotensive effect might involve nitric oxide (NO)-mediated vasodilation, and improvement of endothelial function. In this way, the dietary intake of chlorogenic acids may hold promise for providing a non-pharmacological approach for the prevention and treatment of high BP [42].

Increased concentrations of total plasma homocysteine have been associated with an increased risk of cardiovascular disease [6,41]. Therefore, modifiable factors can influence homocysteine levels. Besides folate intake i.e., the most important dietary determinant of plasma total homocysteine concentration, coffee consumption has an effect on total homocysteine levels in the general population [44].

A positive association, in a dose-dependent manner between homocysteine concentrations and coffee consumption, was reported in a cross-sectional study [44] and has confirmed in randomized controlled trials [45,46]. Grubben et al. [45] have suggested that 10% higher concentration of homocysteine is associated with very high intake of unfiltered coffee (1 L/day), but it is not clear whether the serum lipid fraction, i.e., cholesterol-raising diterpenes present exclusively in unfiltered coffee, is the only factor responsible for the increase in homocysteine concentration. Although the authors were not able to conclude whether this association depends on the brewing method, it seems that unfiltered coffee is more likely to increase total homocysteine than filtered coffee. They speculated that the effect of coffee, mediated by caffeine, on increased plasma homocysteine concentrations could be due to a decrease in blood vitamin B6 concentration, a vitamin related to homocysteine metabolism, whose deficit results in higher production of homocysteine. Additionally, chlorogenic acid, a polyphenol that is present in coffee, may also be partly responsible for the increase of the homocysteine production through increased methylation reactions [47].

On the other hand, moderate coffee consumption among healthy subjects did not significantly increase the homocysteine concentration [48], and a population based-study described that coffee was no longer associated with plasma homocysteine after adjustment for plasma folate concentration [49]. In addition, Mursu et al. [50] found similar results and showed that the consumption of filtered coffee has neither short- nor long-term detectable effects on lipid peroxidation nor on plasma homocysteine concentrations in healthy non-smoking men. More recently, according to Corrêa et al. [51] in Brazilian population, no changes were observed for plasma total homocysteine, after the consumption of three or four cups of paper-filtered coffee per day. The inconsistencies between the above-mentioned epidemiological studies suggest that not all types of coffee brew have the same effect on plasma homocysteine concentrations or that the effect is spurious.

In the current study, we reported that individuals who were consuming more than three cups of filtered coffee per day had lower odds (even not significant) for hyperhomocysteinemia, and moderate consumption of filtered coffee and the polyphenols intake from coffee were inversely associated with hyperhomocysteinemia. A possible hypothesis for this finding is that caffeic acid inhibited hyperhomocysteinemia, elicited leukocyte rolling and adhesion, decreased reactive oxygen species production and activation of cyclooxygenase-2 (COX-2) in endothelial cells. Additionally, caffeic acid was seen to reduce the expression of adhesion molecules such as E-selectin and ICAM-1 on endothelium and integrin CD11b/CD18 (Mac-1 (macrophage-1 antigen)) on leukocytes caused by hyperhomocysteinemia [52,53]. However, the biological mechanisms involved on the effect of coffee consumption on homocysteine concentrations are still unclear and need further investigation.

In addition, coffee consumption has also been associated with alterations in circulating lipids, particularly higher serum TC and LDL-c concentrations, in some observational studies, but not in all [5,6,41]. There are two distinct reasons for these findings. The reason in favor of coffee consumption is that the antioxidants included in coffee might reduce lipid oxidation. The topic has been insufficiently investigated, with two small studies reporting protective (unfiltered coffee for one week) and neutral (filtered coffee for three weeks) effects [6]. The unfavorable reason is that unfiltered coffee is rich in the cholesterol-raising oils (diterpenes, kahweol and cafestol), which contribute significantly to the increase in TC, LDL-c and TG [54]. In contrast to unfiltered coffee, consumption of filtered coffee had no substantial effects on blood lipids [41,55], because the brewing releases oil droplets containing diterpenes from ground coffee beans, and the oil is retained by a paper filter [6,41]. In the present study, we found no association between coffee consumption and serum lipids (cholesterol profiles, and triglyceride levels), perhaps due to the fact that the traditional brewing method of coffee in Brazil is filtering. Thus, our study corroborated and supported this information.

To our knowledge, this is the first study in Brazil to investigate the association between usual coffee consumption and its polyphenols and the main cardiovascular risk factors, and the most important merit of our study is the emphasis of the role of moderate coffee consumption through its relation with BP and homocysteine in decreasing risk of CVD.

However, some limitations of this study should be considered when interpreting results. Firstly, our study is of a cross-sectional nature, which does not allow definitive establishment of causal inference. Furthermore, several confounders in multivariate models were controlled, but other unknown or unmeasured confounders (such as genetic information) may exist. This should be the focus of future studies. Further research, especially coming from randomized clinical trials, is warranted to confirm our findings and establish the biological basis of coffee’s potential preventive effects on CVD.

## 5. Conclusions

Our study shows that moderate coffee consumption and its polyphenols were associated with lower odds of elevated SBP and DBP, and hyperhomocysteinemia in this population. Therefore, moderate coffee consumption, a polyphenol-rich beverage, could provide beneficial effects against clinical cardiovascular risk factors.

## Figures and Tables

**Table 1:** General characteristics of the Health Survey of São Paulo (ISA-Capital) population according to category of coffee consumption. São Paulo, Brazil, 2008/09.

Characteristics	Coffee Consumption, Cups per Day				*p*-Value ^a^
	<1	1–3	≥3	Total	
**No. of subjects**	193	185	179	557	
**Sociodemographic**					
Age (years), median (IQR)	40 (29.0, 53.0)	44 (33.0, 57.0)	47 (36.0, 57.0)	61 (44.0, 70.5)	0.013 ^1^
Sex, *n* (%)					
Male	64 (40.9)	71 (50.2)	71 (47.0)	206 (45.8)	0.360 ^2^
Female	129 (59.1)	114 (49.8)	108 (53.0)	351 (54.2)	
Race, *n* (%)					
White	120 (63.0)	109 (58.1)	117 (62.1)	346 (61.1)	0.918 ^2^
Black	14 (5.8)	18 (6.5)	9 (6.4)	41 (6.2)	
Others	59 (31.2)	58 (35.4)	53 (31.5)	170 (32.7)	
Household *per capita* income, *n* (%)					
<1 MW	80 (34.6)	83 (37.5)	68 (38.3)	231 (36.7)	0.851 ^2^
≥1 MW	113 (65.4)	102 (62.5)	111 (61.7)	326 (63.3)	
Physical activity level, *n* (%)					
Low	157 (74.1)	161 (80.5)	152 (79.2)	470 (77.7)	0.608 ^2^
Moderate/High	36 (25.9)	23 (19.5)	27 (20.8)	86 (22.3)	
Smoking status, *n* (%)					
Non-smoker	114 (65.6)	96 (56.1)	97 (52.1)	307 (58.4)	0.034 ^2^
Smoker	79 (34.4)	82 (43.9)	89 (47.9)	250 (41.6)	
Body Mass Index (kg/m^2^), median (IQR)	25.2 (23.2, 29.1)	24.9 (22.7, 28.6)	26.7 (23.7, 30.8)	26.5 (23.6, 30.5)	0.307 ^1^
**Biochemical**					
SBP (mm Hg), *n* (%)					
Normal	133 (80.1)	130 (87.2)	119 (77.3)	382 (81.5)	0.089 ^2^
Elevated	60 (19.9)	55 (12.8)	60 (22.7)	175 (18.5)	
DBP (mm Hg), *n* (%)					
Normal	163 (85.3)	168 (91.5)	144 (81.8)	465 (86.2)	0.045 ^2^
Elevated	30 (14.7)	27 (8.5)	35 (18.2)	92 (13.8)	
FPG (mg/dL), *n* (%)					
Normal	167 (90.9)	154 (86.0)	158 (92.5)	479 (89.8)	0.177 ^2^
Elevated	26 (9.1)	31 (14.0)	21 (7.4)	78 (10.2)	
TC (mg/dL), *n* (%)					
Normal	118 (69.0)	98 (58.7)	88 (54.0)	304 (61.1)	0.061 ^2^
Elevated	75 (31.0)	87 (41.3)	91 (46.0)	253 (38.9)	
LDL-c (mg/dL), *n* (%)					
Normal	63 (36.2)	46 (26.6)	31 (20.5)	140 (28.2)	0.098 ^2^
Elevated	130 (63.8)	139 (73.4)	148 (79.5)	417 (71.8)	
HDL-c (mg/dL), *n* (%)					
Normal	150 (68.7)	133 (61.1)	129 (61.2)	412 (64.0)	0.466 ^2^
Elevated	43 (31.3)	52 (38.9)	50 (38.8)	145 (36.0)	
TG (mg/dL), *n* (%)					
Normal	135 (74.2)	123 (69.5)	116 (66.8)	374 (70.4)	0.565 ^2^
Elevated	58 (25.8)	62 (30.5)	63 (33.2)	183 (29.6)	
Homocysteine (µmol/L)					
Normal	163 (88.0)	162 (93.4)	156 (89.9)	481 (90.3)	0.350 ^2^
Elevated	30 (12.0)	23 (6.6)	23 (10.1)	76 (9.7)	
**Dietetic**					
Coffee polyphenol intake (mg/day), median (IQR)	66.6 (0, 147.4)	261.6 (225.3, 297.3)	408.4 (351.8, 546.1)	247.0 (145.9, 346.7)	<0.001 ^1^
Caffeine intake (mg/day), median (IQR)	44.7 (24.0, 67.1)	91.3 (80.2, 100.6)	147.3 (117.3, 173.3)	92.4 (60.8, 125.2)	<0.001 ^1^
Alcohol intake (g/day), median (IQR)	0.1 (0.0, 1.2)	0.2 (0.0, 2.3)	0.3 (0.0, 3.2)	0.2 (0.0, 2.1)	0.412 ^1^
Sodium intake (mg/day), median (IQR)	2986.1 (2526.5, 3848.9)	3260.0 (2563.4, 3895.6)	3015.9 (2567.4, 3677.5)	2863.4 (2342.0, 3472.7)	0.995 ^1^
Total energy intake (kcal/day), median (IQR)	1679.4 (1354.4, 2013.8)	1712.8 (1382.3, 2152.6)	1671.1 (1361.8, 2010.2)	1543.9 (1243.8, 1887.9)	0.615 ^1^

Abbreviations: DBP: Diastolic blood pressure; FPG: Fasting plasma glucose; HDL-c: High-density lipoprotein cholesterol; IQR: Interquartile Range; LDL-c: Low-density lipoprotein cholesterol; MW: Minimum Wage; SBP: Systolic blood pressure; TC: Total cholesterol; TG: Triglyceride. Comparisons across categories were performed using the ^1^ Kruskall-Wallis test; ^2^ chi-squared. ^a^ *p*-value < 0.05 was considered statistically significant. The sample weight was considered for statistical analysis.

**Table 2:** Association between cardiovascular risk factors and categories of coffee consumption in Health Survey of São Paulo (ISA-Capital) population. São Paulo, Brazil, 2008/09.

Cardiovascular Risk Factors	Coffee Consumption, cups per Day		
	<1	1–3	≥3
**Elevated SBP** (≥140 mm Hg)			
OR crude (unadjusted)	1.00	0.58 (0.33, 1.05)	1.17 (0.63, 2.21)
OR adjusted ^1^	1.00	0.45 (0.26, 0.78)	0.81 (0.41, 1.61)
**Elevated DBP** (≥90 mm Hg)			
OR crude (unadjusted)	1.00	0.54 (0.24, 1.22)	1.30 (0.66, 2.54)
OR adjusted ^1^	1.00	0.44 (0.20, 0.98)	0.89 (0.45, 1.75)
**Increased FPG** (≥100 mg/dL)			
OR crude (unadjusted)	1.00	1.64 (0.82, 3.24)	0.81 (0.34, 1.92)
OR adjusted ^2^	1.00	1.39 (0.60, 3.23)	0.72 (0.22, 2.26)
**Increased TC** (≥200 mg/dL)			
OR crude (unadjusted)	1.00	1.57 (0.86, 2.85)	1.89 (1.22, 2.93)
OR adjusted ^3^	1.00	1.46 (0.76, 2.80)	1.45 (0.94, 2.22)
**Increased LDL-c** (>100 mg/dL)			
OR crude (unadjusted)	1.00	1.56 (0.76, 3.20)	2.20 (1.12, 4.32)
OR adjusted ^3^	1.00	1.46 (0.68, 3.12)	2.07 (0.92, 4.67)
**Reduced HDL-c** (<40 mg/dL men; <50 mg/dL women)			
OR crude (unadjusted)	1.00	1.40 (0.77, 2.54)	1.39 (0.75, 2.59)
OR adjusted ^3^		1.67 (0.87, 3.20)	1.77 (0.80, 3.94)
**Increased TG** (≥150 mg/dL)			
OR crude (unadjusted)	1.00	1.26 (0.59, 2.71)	1.43 (0.73, 2.80)
OR adjusted ^3^	1.00	1.26 (0.59, 2.63)	1.35 (0.61, 2.98)
**Increased Homocysteine** (>12 µmol/L adults; >16 µmol/L older adults)			
OR crude (unadjusted)	1.00	0.52 (0.16, 1.43)	0.82 (0.35, 1.94)
OR adjusted ^4^	1.00	0.32 (0.11, 0.93)	0.43 (0.19, 1.01)

Odds ratio (OR) and 95% Confidence Interval (95% CI) were calculated by using multivariate logistic regression. Models were adjusted for age, sex, race, body mass index (BMI), smoking, alcohol, physical activity, household per capita income, intake of caffeine, added sugars, total energy intake, and saturated fat: ^1^ additionally adjusted for sodium intake, and antihypertensive drugs; ^2^ additionally adjusted for hypoglycemic drugs; ^3^ additionally adjusted for monounsaturated fat, polyunsaturated fat, and hypolipidemic drugs; ^4^ additionally adjusted for vitamins folate, B6, and B12. Abbreviations: DBP: Diastolic blood pressure; FPG: Fasting plasma glucose; HDL-c: High-density lipoprotein cholesterol; LDL-c: Low-density lipoprotein cholesterol; SBP: Systolic blood pressure; TC: Total cholesterol; TG: Triglyceride.

**Table 3:** Association between cardiovascular risk factors and categories of coffee polyphenol intake in Health Survey of São Paulo (ISA-Capital) population. São Paulo, Brazil, 2008/09.

Cardiovascular Risk Factors	Polyphenol Intake from Coffee, mg per Day ^a^		
	<101	101–337	≥337
**Elevated SBP** (≥140 mm Hg)			
OR crude (unadjusted)	1.00	0.55 (0.30, 1.02)	0.94 (0.52, 1.73)
OR adjusted ^1^	1.00	0.46 (0.24, 0.87)	0.72 (0.35,1.45)
**Elevated DBP** (≥90 mm Hg)			
OR crude (unadjusted)	1.00	0.52 (0.26, 1.06)	0.86 (0.45, 1.65)
OR adjusted ^1^	1.00	0.51 (0.26, 0.98)	0.70 (0.39, 1.27)
**Increased FPG** (≥100 mg/dL)			
OR crude (unadjusted)	1.00	1.77 (0.97, 3.23)	0.66 (0.28, 1.58)
OR adjusted ^2^	1.00	1.98 (0.87, 4.54)	0.71 (0.23, 2.20)
**Increased TC** (≥200 mg/dL)			
OR crude (unadjusted)	1.00	0.96 (0.54, 1.72)	1.35 (0.92, 1.98)
OR adjusted ^3^	1.00	0.83 (0.44, 1.57)	1.07 (0.71, 1.59)
**Increased LDL-c** (>100 mg/dL)			
OR crude (unadjusted)	1.00	1.05 (0.49, 2.25)	1.80 (0.86, 3.75)
OR adjusted ^3^	1.00	0.99 (0.45, 2.17)	1.67 (0.70, 4.03)
**Reduced HDL-c** (<40 mg/dL men; <50 mg/dL women)			
OR crude (unadjusted)	1.00	1.31 (0.78, 2.18)	1.31 (0.74, 2.31)
OR adjusted ^3^	1.00	1.27 (0.70, 2.31)	1.51 (0.74, 3.07)
**Increased TG** (≥150 mg/dL)			
OR crude (unadjusted)	1.00	0.87 (0.45, 1.71)	1.00 (0.52, 1.92)
OR adjusted ^3^	1.00	0.72 (0.36, 1.45)	0.86 (0.41, 1.78)
**Increased Homocysteine** (>12 µmol/L adults; >16 µmol/L older adults)			
OR crude (unadjusted)	1.00	0.52 (0.18, 1.51)	0.84 (0.40,1.81)
OR adjusted ^4^	1.00	0.29 (0.11, 0.78)	0.59 (0.31, 1.11)

Odds ratio (OR) and 95% CI were calculated by using multivariate logistic regression. Models were adjusted for age, sex, race, BMI, smoking, alcohol, physical activity, household per capita income, intake of caffeine, added sugars, total energy intake, saturated fat, and other polyphenol intake (except polyphenols from coffee). ^1^ additionally adjusted for sodium intake, and antihypertensive drugs; ^2^ additionally adjusted for hypoglycemic drugs; ^3^ additionally adjusted for monounsaturated fat, polyunsaturated fat, and hypolipidemic drugs; ^4^ additionally adjusted for vitamins folate, B6, and B12. ^a^ < 101 mg/day (corresponding to <1 cup coffee/day), 101–337 mg/day (corresponding to 1–3 cups/day), and ≥ 337 mg/day (corresponding to ≥3 cups/day). Abbreviations: DBP: Diastolic blood pressure; FPG: Fasting plasma glucose; HDL-c: High-density lipoprotein cholesterol; LDL-c: Low-density lipoprotein cholesterol; SBP: Systolic blood pressure; TC: Total cholesterol; TG: Triglyceride.

## References

[1] Mozaffarian D., Benjamin E.J., Go A.S., Amett D.K., Blaha M.J., Cushman M., Dai S., De Simone G., Ferguson T.B., Ford E. (2015). Heart disease and stroke statistics-2015 update: A report from the American Heart Association. Circulation.

[2] World Health Organization (WHO) (2011). Global Atlas on Cardiovascular Disease Prevention and Control.

[3] International Coffee Organization Trade Statistics. http://www.ico.org/profiles_e.asp.

[4] Souza A.M., Pereira R.A., Yokoo E.M., Levy R.B., Sichieri R. (2013). Alimentos mais consumidos no Brasil: Inquérito Nacional de Alimentação 2008–2009 (Most consumed foods in Brazil: National Dietary Survey 2008–2009). Rev. Saúde Publica.

[5] O’Keefe J.H., Bhatti S.K., Patil H.R., DiNicolantonio J.J., Lucan S.C., Lavie C.J. (2013). Effects of habitual coffee consumption on cardiometabolic disease, cardiovascular health, and all-cause mortality. J. Am. Coll. Cardiol..

[6] Cano-Marquina A., Tarín J.J., Cano A. (2013). The impact of coffee on health. Maturitas.

[7] Ding M., Bhupathiraju S.N., Chen M., van Dam R.M., Hu F.B. (2014). Caffeinated and Decaffeinated Coffee Consumption and Risk of Type 2 Diabetes: A systematic review and a dose-response meta-analysis. Diabetes Care.

[8] Noordzij M., Uiterwaal C.S.P.M., Arends L.R., Kok F.J., Grobbee D.E., Geleijnse J.M. (2005). Blood pressure response to chronic intake of coffee and caffeine: A meta-analysis of randomized controlled trials. J. Hypertens..

[9] Mesas A.E., Leon-Muñoz L.M., Rodriguez-Artalejo F., Lopez-Garcia E. (2011). The effect of coffee on blood pressure and cardiovascular disease in hypertensive individuals: A systematic review and meta-analysis. Am. J. Clin. Nutr..

[10] Rodrigues I.M., Klein L.C. (2006). Boiled or filtered coffee? Effects of coffee and caffeine on cholesterol, fibrinogen and C-reactive protein. Toxicol. Rev..

[11] Whitehead N., White H. (2013). Systematic review of randomized controlled trials of the effects of caffeine or caffeinated drinks on blood glucose concentrations and insulin sensitivity in people with diabetes mellitus. J. Hum. Nutr. Diet..

[12] Wu J.N., Ho S.C., Zhou C., Ling W.H., Chen W.Q., Wang C.L., Chen Y.-M. (2009). Coffee consumption and risk of coronary heart diseases: A meta-analysis of 21 prospective cohort studies. Int. J. Cardiol..

[13] Ding M., Satija A., Bhupathiraju S.N., Hu Y., Sun Q., Han J., Lopez-Garcia E., Willett W., van Dam R.M., Hu F.B. (2015). Association of Coffee Consumption with Total and Cause-Specific Mortality in Three Large Prospective Cohorts. Circulation.

[14] Ding M., Bhupathiraju S.N., Satija A., van Dam RM., Hu F.B. (2014). Long-term coffee consumption and risk of cardiovascular disease: A systematic review and a dose-response meta-analysis of prospective cohort studies. Circulation.

[15] Guo X., Tresserra-Rimbau A., Estruch R., Martínez-González M., Medina-Remón A., Castañer O., Corella D., Salas-Salvadó J., Lamuela-Raventós R.M. (2016). Effects of Polyphenol, Measured by a Biomarker of Total Polyphenols in Urine, on Cardiovascular Risk Factors After a Long-Term Follow-Up in the PREDIMED Study. Oxid. Med. Cell. Longev..

[16] Kempf K., Herder C., Erlund I., Kolb H., Martin S., Carstensen M., Koenig W., Sundvall J., Bidel S., Kuha S. (2010). Effects of coffee consumption on subclinical inflammation and other risk factors for type 2 diabetes: A clinical trial. Am. J. Clin. Nutr..

[17] Natella F., Nardini M., Giannetti I., Dattilo C., Scaccini C. (2002). Coffee drinking influences plasma antioxidant capacity in humans. J. Agric. Food Chem..

[18] Fuentes E., Palomo I. (2014). Mechanisms of endothelial cell protection by hydroxycinnamic acids. Vasc. Pharmacol..

[19] Grosso G., Micek A., Godos J., Sciacca S., Pajak A., Martínez-González M.A., Giovannucci E.L., Galvano F. (2016). Coffee consumption and risk of all-cause, cardiovascular, and cancer mortality in smokers and non-smokers: A dose-response meta-analysis. Eur. J. Epidemiol..

[20] Guenther P.M., DeMaio T.J., Ingwersen L.A., Berline M. The multiple-pass approach for the 24 h recall in the Continuing Survey of Food Intakes by Individuals (CSFII) 1994 ± 1996. Proceedings of the International Conference on Dietary Assessment Methods.

[21] Blanton C.A., Moshfegh A.J., Baer D.J., Kretsch M.J. (2006). The USDA Automated Multiple-Pass Method accurately estimates group total energy and nutrient intake. J. Nutr..

[22] Harttig U., Haubrock J., Knueppel S., Boeing H., Consortium EFCOVAL (2011). The MSM program: Web-based statistics package for estimating usual dietary intake using the Multiple Source Method. Eur. J. Clin. Nutr..

[23] Scalbert A. Phenol-Explorer: Database on Polyphenol Content in Foods. http://phenol-explorer.eu/.

[24] Pérez-Jiménez J., Neveu V., Vos F., Scalbert A. (2010). Systematic analysis of the content of 502 polyphenols in 452 foods and beverages: An application of the Phenol-Explorer database. J. Agric. Food Chem..

[25] Miranda A.M., Steluti J., Fisberg R.M., Marchioni D.M. (2016). Dietary intake and food contributors of polyphenols in adults and elderly adults of Sao Paulo: A population-based study. Br. J. Nutr..

[26] Craig C.L., Marshall A.L., Sjostrom M., Bauman A.E., Booth M.L., Ainsworth B.E., Pratt M., Ekelund U., Yngve A., Sallis J.F. (2003). International physical activity questionnaire: 12-country reliability and validity. Med. Sci. Sports Exerc..

[27] The IPAQ Group International Physical Activity Questionnaire. http://www.ipaq.ki.se.

[28] World Health Organization (WHO) (1995). Physical Status: The Use E Interpretation of Anthropometry.

[29] Sociedade Brasileira de Cardiologia, Sociedade Brasileira de Hipertensão, Sociedade Brasileira de Nefrologia (2007). V Diretrizes brasileiras de hipertensão arterial. Arq. Bras. Cardiol..

[30] Chobanian A.V., Bakris G.L., Black H.R., Cushman W.C., Green L.A., Izzo J.L. (2003). The Seventh Report of the Joint National Committee on Detection, Evaluation, and Treatment of High Blood Pressure. The JNC 7 Report. JAMA.

[31] Expert Dyslipidemia Panel of the International Atherosclerosis Society (2014). An International Atherosclerosis Society Position Paper: Global recommendations for the management of dyslipidemia-full report. J. Clin. Lipidol..

[32] Refsum H., Smith A.D., Ueland P.M., Nexo E., Clarke R., McPartlin J., Johnston C., Engbaek F., Schneede J., McPartlin C. (2004). Facts and recommendations about total homocysteine determinations: An expert opinion. Clin. Chem..

[33] Butt M.S., Sultan M.T. (2011). Coffee and its consumption: Benefits and risks. Crit. Rev. Food Sci. Nutr..

[34] Liu J., Sui X., Lavie C.J., Hebert J.R., Earnest C.P., Zhang J., Blair S.N. (2013). Association of coffee consumption with all-cause and cardiovascular disease mortality. Mayo Clin. Proc..

[35] Grioni S., Agnoli C., Sieri S., Pala V., Ricceri F., Masala G., Saieva C., Panico S., Mattiello A., Chiodini P. (2015). Espresso coffee consumption and risk of coronary heart disease in a large Italian Cohort. PLoS ONE.

[36] Floegel A., Pischon T., Bergmann M.M., Teucher B., Kaaks R., Boeing H. (2012). Coffee consumption and risk of chronic disease in the European Prospective Investigation into Cancer and Nutrition (EPIC)-Germany study. Am. J. Clin. Nutr..

[37] Lopez-Gracia E., Van Dam R.M., Willett W.C., Rimm E.B., Manson J.E., Stampfer M.J., Rexrode K.M., Hu F.B. (2006). Coffee consumption and coronary heart disease in men and women. Circulation.

[38] Riksen N.P., Rongen G.A., Smits P. (2009). Acute and long-term cardiovascular effects of coffee: Implications for coronary heart disease. Pharmacol. Ther..

[39] Echeverri D., Montes F.R., Cabrera M., Galán A., Prieto A. (2010). Caffeine’s vascular mechanisms of action. Int. J. Vasc. Med..

[40] Godos J., Pluchinotta F.R., Marventano S., Buscemi S., Li Volti G., Galvano F., Grosso G. (2014). Coffee components and cardiovascular risk: Beneficial and detrimental effects. Int. J. Food Sci. Nutr..

[41] Ranheim T., Halvorsen B. (2005). Coffee consumption and human health-beneficial or detrimental?-Mechanisms for effects of coffee consumption on different risk factors for cardiovascular disease and type 2 diabetes mellitus. Mol. Nutr. Food Res..

[42] Zhao Y., Wang J., Ballevre O., Luo H., Zhang W. (2012). Antihypertensive effects and mechanisms of chlorogenic acids. Hypertens. Res..

[43] Mubarak A., Bondonno C.P., Liu A.H., Considine M.J., Rich L., Mas E., Croft K.D., Hodgson J.M. (2012). Acute effects of chlorogenic acid on nitric oxide status, endothelial function, and blood pressure in healthy volunteers: A randomized trial. J. Agric. Food Chem..

[44] De Bree A., Verschuren W.M., Blom H.J., Kromhout D. (2001). Lifestyle factors and plasma homocysteine concentrations in a general population sample. Am. J. Epidemiol..

[45] Grubben M.J., Boers G.H., Blom H.J., Broekhuizen R., de Jong R., van Rijt L., de Ruijter E., Swinkels D.W., Nagengast F.M., Katan M.B. (2000). Unfiltered coffee increases plasma homocysteine concentrations in healthy volunteers: A randomized trial. Am. J. Clin. Nutr..

[46] Urgert R.A., van Vliet T., Zock P.L., Katan M.B. (2000). Heavy coffee consumption and plasma homocysteine: A randomized controlled trial in healthy volunteers. Am. J. Clin. Nutr..

[47] Olthof M.R., Hollman P.C., Zock P.L., Katan M.B. (2001). Consumption of high doses of chlorogenic acid, present in coffee, or of black tea increases total homocysteine concentrations in humans. Am. J. Clin. Nutr..

[48] Esposito F., Morisco F., Verde V., Ritieni A., Alezio A., Caporaso N., Fogliano V. (2003). Moderate coffee consumption increases plasma glutathione but not homocysteine in healthy subjects. Aliment. Pharmacol. Ther..

[49] Saw S.M., Yuan J.M., Ong C.N., Arakawa K., Lee H.P., Coetzee G.A., Yu M.C. (2001). Genetic, dietary and other lifestyle determinants of plasma homocysteine concentrations in middle-aged and older Chinese men and women in Singapore. Am. J. Clin. Nutr..

[50] Mursu J., Voutilainen S., Nurmi T., Alfthan G., Virtanen J.K., Rissanen T.H., Happonen P., Nyyssönen K., Kaikkonen J., Salonen R. (2005). The effects of coffee consumption on lipid peroxidation and plasma total homocysteine concentrations: A clinical trial. Free Radic. Biol. Med..

[51] Corrêa T.A., Rogero M.M., Mioto B.M., Tarasoutchi D., Tuda V.L., César L.A., Torres E.A. (2013). Paper-filtered coffee increases cholesterol and inflammation biomarkers independent of roasting degree: A clinical trial. Nutrition.

[52] Zhao H.P., Feng J., Sun K., Liu Y.Y., Wei X.H., Fan J.Y., Huang P., Mao X.-W., Zhou Z., Wang C.-S. (2012). Caffeic acid inhibits acute hyperhomocysteinemia-induced leukocyte rolling and adhesion in mouse cerebral venules. Microcirculation.

[53] Moon M.K., Lee Y.J., Kim J.S., Kang D.G., Lee H.S. (2009). Effect of caffeic acid on tumor necrosis factor-alpha-induced vascular inflammation in human umbilical vein endothelial cells. Biol. Pharm. Bull..

[54] Cai L., Ma D., Zhang Y., Liu Z., Wang P. (2012). The effect of coffee consumption on serum lipids: A meta-analysis of randomized controlled trials. Eur. J. Clin. Nutr..

[55] Rebello S.A., van Dam R.M. (2013). Coffee consumption and cardiovascular health: Getting to the heart of the matter. Curr. Cardiol. Rep..

